# Simultaneous cardiac and respiratory inhibition during seizure precedes death in the DBA/1 audiogenic mouse model of SUDEP

**DOI:** 10.1371/journal.pone.0223468

**Published:** 2019-10-21

**Authors:** William P. Schilling, Morgan K. McGrath, Tianen Yang, Patricia A. Glazebrook, Carl L. Faingold, Diana L. Kunze

**Affiliations:** 1 Rammelkamp Center for Education and Research, MetroHealth Campus, Case Western Reserve University, Cleveland, Ohio, United States of America; 2 Department of Physiology and Biophysics, Case Western Reserve University, Cleveland, Ohio, United States of America; 3 Department of Pharmacology, Southern Illinois University School of Medicine, Springfield, Illinois, United States of America; 4 Department of Neurosciences, Case Western Reserve University, Cleveland, Ohio, United States of America; University of Modena and Reggio Emilia, ITALY

## Abstract

This study was designed to evaluate cardiac and respiratory dysfunction in a mouse model of sudden unexpected death in epilepsy i.e., SUDEP. We simultaneously monitored respiration via plethysmography and the electrocardiogram via telemetry before, during, and after an audiogenic seizure. DBA/1 mice responded to an acoustic stimulus with one or two cycles of circling and jumping before entering a clonic/tonic seizure. This was followed by death unless the mice were resuscitated by mechanical ventilation using room air. During the initial clonic phase, respiration declined and cardiac rhythm is slowed. By the tonic phase, respiration had ceased, atrial P-waves were absent or dissociated from the QRS complex, and heart rate had decreased from 771±11 to 252±16 bpm. Heart rate further deteriorated terminating in asystole unless the mice were resuscitated at the end of the tonic phase which resulted in abrupt recovery of P-waves and a return to normal sinus rhythm, associated with gasping. Interestingly, P-waves were preserved in the mice treated with methylatropine during the pre-ictal period (to block parasympathetic stimulation) and heart rate remained unchanged through the end of the tonic phase (765±8 vs. 748±21 bpm), but as in control, methylatropine treated mice died from respiratory arrest. These results demonstrate that a clonic/tonic seizure in the DBA/1 mouse results in abrupt and simultaneous respiratory and cardiac depression. Although death clearly results from respiratory arrest, our results suggest that seizure activates two central nervous system pathways in this model—one inhibits respiratory drive, whereas the other inhibits cardiac function via vagal efferents. The abrupt and simultaneous recovery of both respiration and cardiac function with mechanical ventilation within an early post-ictal timeframe shows that the vagal discharge can be rapidly terminated. Understanding the central mechanism associated with the abrupt cardiorespiratory dysfunction and equally abrupt recovery may provide clues for therapeutic targets for SUDEP.

## Introduction

Patients with epilepsy refractory to drug treatment are reported to be more susceptible to sudden death than the overall population. In most cases, the cause of death is not known and they are classified as a sudden and unexpected death in epilepsy (SUDEP; see [1] for a review). The underlying mechanisms responsible for SUDEP are poorly understood, but respiratory failure has been seen as the critical factor in most of the observed cases of SUDEP [2]. Substantial clinical data also point to cardiac dysfunction following seizure [3,4]. The decreased cardiac rate and the subsequent arrhythmias leading to transient asystole are often suggested to be the result of hypoxia and hypercapnia produced by the apnea. Although the Mortemus study clearly showed that terminal apnea always precedes the terminal asystole [3], the relationship between respiratory and cardiac dysfunction remains unclear.

DBA mice have been employed as a model for SUDEP [1, 5–8]. These mice develop a generalized clonic-tonic seizure in response to an audiogenic stimulus. The seizure results in immediate respiratory arrest and death unless the mouse is rescued within 5–10 seconds post-ictally by mechanical ventilation. Consistent with human data, previous studies in DBA/1 mice point to seizure-initiated autonomic dysfunction as the critical initiating cause of death, specifically respiratory arrest [8]. Previous studies have also suggested that seizure causes profound bradycardia in the DBA mouse, but as in human patients, the relationship between respiratory arrest and bradycardia in this mouse model is unclear. Indeed, seizure may initiate not only respiratory depression, but also activation of vagal discharge leading to cardiac dysrhythmia and death [9–11]. In the present study, we used simultaneous recordings of respiration, cardiac rhythm and behavior to examine the timing of the respiratory depression, and cardiac dysrhythmia during seizure. We found that cardiac inhibition with characteristics of sino-atrial (SA) and atrial-ventricular (AV) nodal suppression occurs at the same time as the apnea. This has important consequences for the way in which future studies are designed to clarify mechanisms and introduce treatments.

## Methods

### Animals

All animal-use protocols were reviewed and approved for ethical practice by the Institutional Animal Care and Use Committee (IACUC) of Case Western Reserve University (Current approved protocol #2018–0012). As described below, DBA/1 mice, which are a model for SUDEP, die during or within seconds of experiencing a generalized clonic-tonic seizure. Death occurs by respiratory arrest with concomitant cardiac dysfunction. There is a very brief post-ictal time period (5–10 sec) when the mouse can be resuscitated (see below), otherwise death quickly occurs. In the experiments evaluating the effects of methylatropine, we determined survival. Consequently, 7 control mice and 4 methylatropine-treated mice were allowed to die without intervention. Euthanasia is not used after seizure for the following reason. The critical electrocardiogram (ECG) and respiratory data that we are collecting occurs during the clonic/tonic phase of the seizure (which lasts approximately 10 sec) and during the early post-ictal period (approximately 5–10 more seconds). We are collecting these data because it is this period that correlates best with the clinical phenotype of SUDEP in patients. It is important to note that during these 15–20 seconds, respiration ceases, cardiac rhythm is blocked and the EEG has flatlined. Thus, there is no benefit to intervening with euthanasia. It is important to note that seizures induced by acoustic stimuli do not result in pain, since relatively long-lasting analgesia results from this type of seizure [12]. The IACUC specifically reviewed and approved the mortality aspects of this protocol.

This study involved a total of 50 DBA/1 male mice obtained from Jackson Laboratory (Bar Harbor, ME) or Envigo (Indianapolis, IN). No differences were seen in the seizure responses of the mice from these two vendors. The animals used solely for respiratory studies were subjected to the standard priming protocol of 3–4 seizures before age of 30 days [8]. The animals used for ECG studies were implanted with transmitter at 4–7 weeks, i.e, when they reached a weight of 18–20 grams. Following recovery from implantation, the mice were subjected to the priming protocols.

### Telemetry

DBA/1 mice were instrumented with transmitters (ETAF10; from Data Sciences International (DSI), St. Paul, MN) for ECG recording with leads positioned in a modified lead 2 configuration as previously described [10]. Data acquisition and analysis was performed using Ponemah (DSI, St Paul, MN) or AD Instruments Power Lab software (Colorado Springs, CO). The ECG data were digitized at 1 KHz. Respiratory data were digitized at 250 Hz.

### Seizure induction and plethysmography

The mice were placed in a closed, air-tight, circular, Perspex plethysmography chamber (Data Sciences International (DSI); 18 cm diameter) to monitor respiration. Prior to each recording, air volumes were calibrated following instructions provided by the manufacturer (DSI). Respiration was continuously recorded using Ponemah software (DSI). Simultaneously, the ECG was transmitted to a receiver placed under the circular chamber. Five-to-ten minutes of pre-seizure control data were obtained after which an acoustic stimulus was delivered from a bell (Piezo Siren, Radio Shack 273–079; intensity broad band stimulus ≥ 108 dB) affixed to the lid inside the chamber until the mouse underwent seizure, but no longer than 60 sec. Video recording allowed correlation of the seizure behaviors with the ECG and respiratory data.

As mentioned in the *Introduction*, the DBA/1 mice can be resuscitated by mechanical ventilation with room air. This was accomplished by quickly opening the plethysmography chamber and applying positive pressure ventilation using a Harvard Rodent Respirator (Model 683) at a rate of 145 cycles/min via tubing placed loosely over the nose of the mouse. When the animal was clearly breathing independently, generally within 5–15 sec of starting resuscitation (13 ± 2.4 sec, n = 12) the chamber was closed and recording of respiration resumed. The ECG was continuously recorded during the resuscitation period by holding the mouse near to the bottom of the chamber (i.e., near the recording pad) while applying the ventilator tubing to the nose. In all figures bpm refers to “breaths per minute” or “beats per minute” when discussing respiration or cardiac function, respectively.

### Drugs

A subset of mice were administered methylatropine (0.1, 0.2, or 5mg/kg, ip) 20–30 minutes prior to the audio stimulus to test the effect of muscarinic receptor blockade on cardiac rhythm during the clonic/tonic seizure and in the immediate post-ictal period. Methylatropine bromide was obtained from Sigma-Aldrich.

### Statistical evaluation

Unless otherwise noted in the text, data are presented as mean ± standard error, se. Significant differences were determined where appropriate, using paired Student’s t-test, two sample t-test, or repeated measure ANOVA, followed by the Bonferroni post hoc test; p-values <0.05 were considered significant.

## Results

Every seizure was characterized by the following sequence of events that defined the clonic and tonic phases. Each mouse responded to the auditory stimulus with 1-or-2 cycles of rapid circling and jumping (C/J) followed by a generalized clonic/tonic (C/T) seizure. The beginning of the clonic phase was defined by the loss of upright posture and loss of the righting reflex (i.e, while running in circles within the chamber the mouse falls onto one side). This was followed by hindlimb and/or forelimb treading, and curling or hunching of the back. Within seconds, a sudden tonic extension of the forelimbs and hindlimbs and stiffening of the tail was observed. This sudden extension of the limbs is clearly visible in the video recordings and defines the end of the clonic phase and beginning of the tonic phase. The tonic phase ended with relaxation of the tail and limbs, followed by death. This sequence was highly reproducible and generally was initiated within 30 s of the acoustic stimulus (19 ± 3.4 sec, n = 19).

### Respiratory function

**Fig 1:**
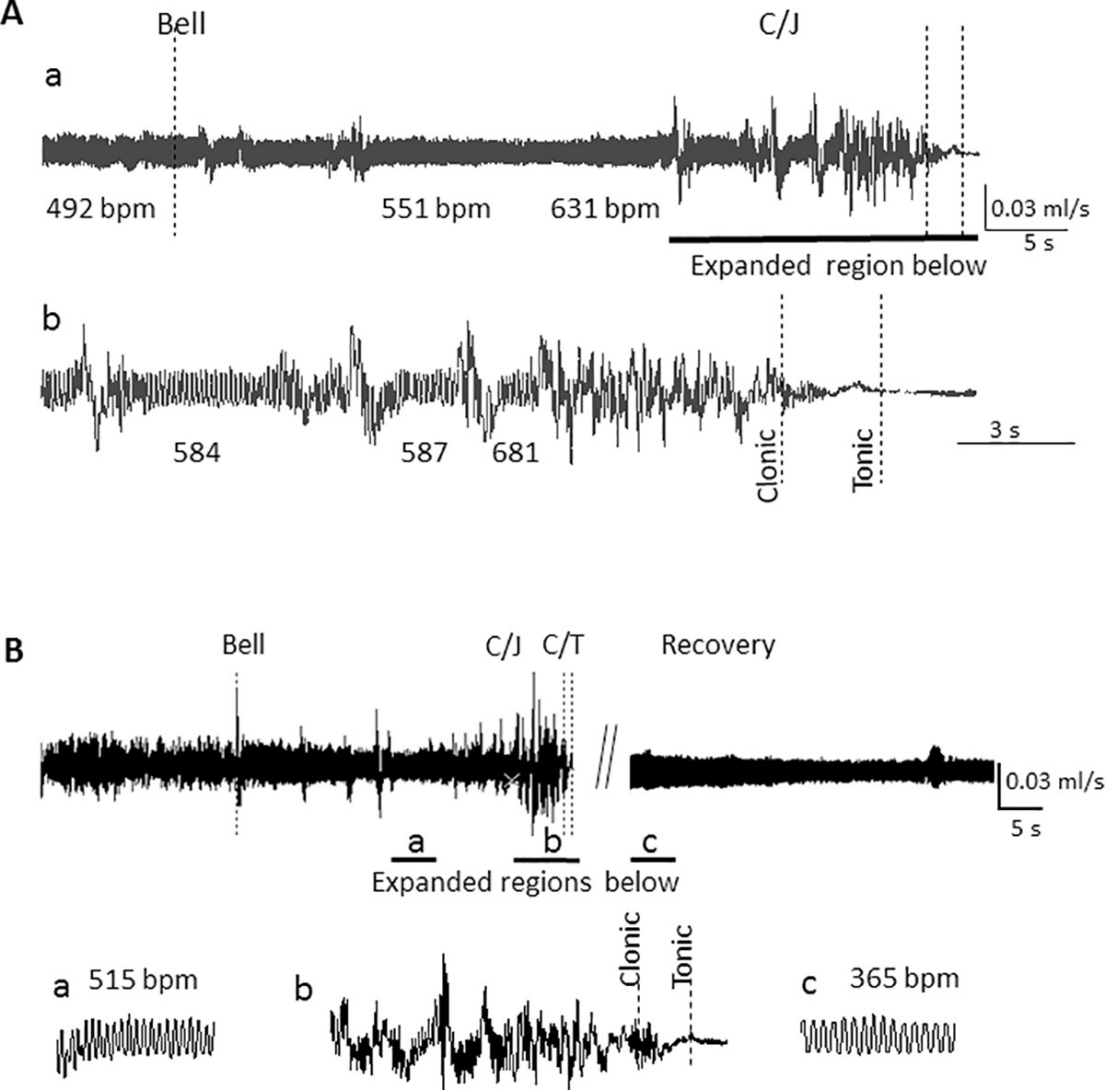
Respiratory arrest occurs during the clonic phase, but before the start of the tonic phase. **A:** Plethysmography recording of respiration is shown during a C/T seizure in the DBA/1 mouse. **a**. Mouse responds to an acoustic stimulation (*Bell*) with 2 cycles of circling and jumping (*C/J*) and a single cycle of circling before entering a clonic/tonic (*C/T*) seizure. As denoted by the breaths per minute (bpm) calculated from random 10 sec samples within the range above the number, respiration frequency varied before the bell, depending on the ongoing activity of the mouse (consistent with exploring, sniffing and grooming). **b.** As shown in the expanded trace, respiration is maintained between cycles of circling/jumping, but is reduced entering the clonic phase and has ceased before entering the tonic phase. Dashed lines in all figures indicate the beginning of the phase indicated by the label. This mouse was not resuscitated. Air flow is expressed as ml/sec in this and subsequent figures. **B:** Respiration recovers following resuscitation by mechanical ventilation. Mouse responds to the stimulus with a single period of circling/jumping followed quickly by the C/T phases. The chamber was opened (*at // marks*) and room air applied through tubing placed loosely over the nose. This mouse responded within 8 sec with a series of visualized gasps that evolved to normal inspiration. When confident the mouse was breathing independently, the chamber was closed and the recording continued. As seen in the expanded traces (**a, b, c**), respiration again ceased by the end of the clonic phase. Following resuscitation **(c)** the animal was breathing independently, but was not physically recovered and remained still with little movement in the chamber. Traces **a** and **c** are 2.5 sec in length; **b** is 8 s.

Previous studies revealed that DBA/1 mice die from seizure-induced respiratory arrest [5–8] but the kinetic relationship between the clonic and tonic phases and respiratory failure is unknown. In our initial experiments, we monitored only respiration before, during, and after seizure to define the exact moment when respiration ceased. Respiration was recorded for at least 5–10 min prior to the delivery of an auditory stimulus. Respiratory rate in the un-anesthetized freely moving DBA/1 mouse varied widely from approximately 160 breaths per minute (bpm) at rest to over 700 bpm during exploration, grooming and sniffing activity, a range similar to previously reported mouse values [13,14]. Presentation of the auditory stimulus (i.e., the bell) initiated one or two cycles of wild circling and was terminated when the mouse progressed thru the C/T seizure. At this time the mouse either died or was resuscitated via mechanical ventilation with room air (see methods). An example of raw respiratory data from one of 4 mice that were not resuscitated is shown in Fig 1A. Although movement during the cycles of rapid circling, and the accompanied jumping, interfered with the plethysmography, it is evident in this figure that respiration was maintained in the periods between the cycles of circling/jumping (C/J). As these data demonstrate in the expanded trace, respiratory amplitude and frequency declined rapidly as the animal entered the clonic phase to the point of cessation before tonic limb extension (Fig 1A, b).

Fifteen mice were resuscitated at the end of the tonic phase. The respiratory recording from one mouse is shown in Fig 1B with expanded sections shown below (a, b, c). As in Fig 1A, the respiration in this mouse ceased during the clonic phase. In 12 of 15 mice, 2 cycles of circling and jumping were observed, whereas the other 3 mice had only one cycle. The respiratory rate for the 12 mice was 561±17 before the first period of C/J, and was 526±15 bpm between episodes of C/J; these were not significantly different (p>0.11, Student’s t-test). The recorded respiratory deflections generally disappeared about halfway through the clonic phase (58 ± 5%, range 36–90%, n = 12). The lapse of time before initiation of resuscitation (measured from the beginning of the tonic phase) ranged from 4 to 10 sec with mean of 6.1 ± 0.4 sec. With ventilation, respiration returned with a series of gasps (recognized in video recordings) that evolved into a regular and stable rate at which time the chamber was closed and recording continued. In 10/15 mice where it was possible to identify initial spontaneous respiratory movements on video, the time to recovery after ventilation was applied was 9.9± 0.9 sec.

### Cardiac function

**Fig 2:**
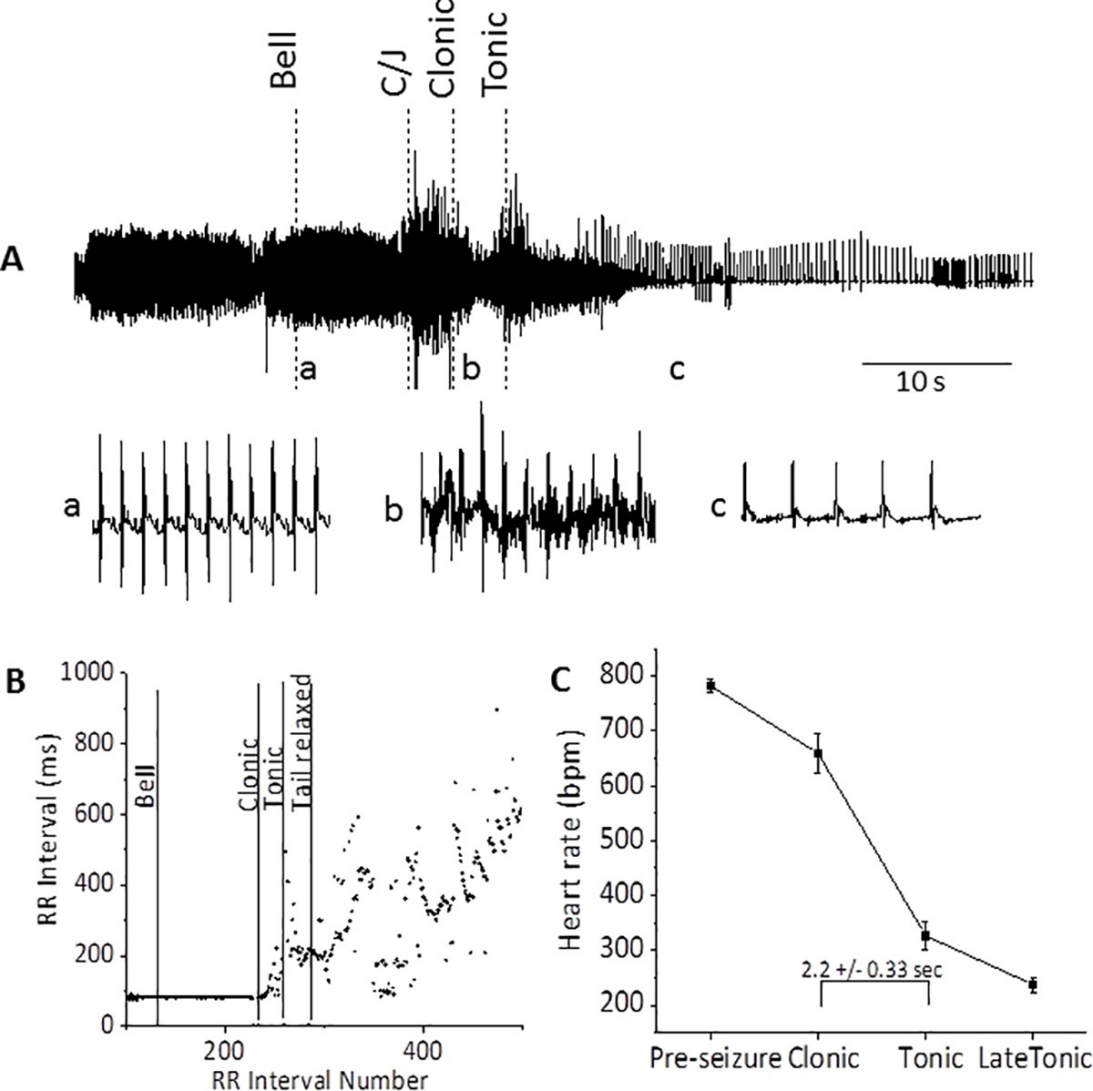
Bradycardia abruptly begins during the clonic phase and rapidly progresses to a slow ventricular rhythm. **A**: A telemetry recording of the ECG is shown during a C/T seizure. As indicated below the trace, one sec samples are expanded from selected regions; just after the bell (**a**), early in the clonic period (**b**), and late the tonic period (**c**) when the seizure activity has ended. The ECG during the circling/jumping (*C/J*) and clonic phases is partially obscured by electrical activity of muscle contraction. However, regions of the ECG during the seizure provide clear delineation of the RR-interval as shown in expanded trace **b**. In this typical example, the ECG lacks P-waves following seizure (trace **c**), indicating suppression of the SA nodal rhythm, and a pronounced bradycardia characteristic of AV-block with a ventricular escape rhythm. This mouse was not resuscitated. **B:** The figure shows a continuous diary of the RR-interval (tachogram) from the recording in A; note the abrupt transition to irregular rhythm during the clonic phase. The RR-interval in the post-ictal period increased rapidly and reflects a variable and progressive slowing of the ventricular rate. Dashed lines indicate the beginning of the phase indicated by the label. **C**: Mean heart rate at each of the periods indicated on the X-axis was quantified from the R-R intervals. Rate was calculated over 10 sec with the exception of the short clonic period which was on average 2.2 ± 0.33 sec (n = 6).

**Fig 3:**
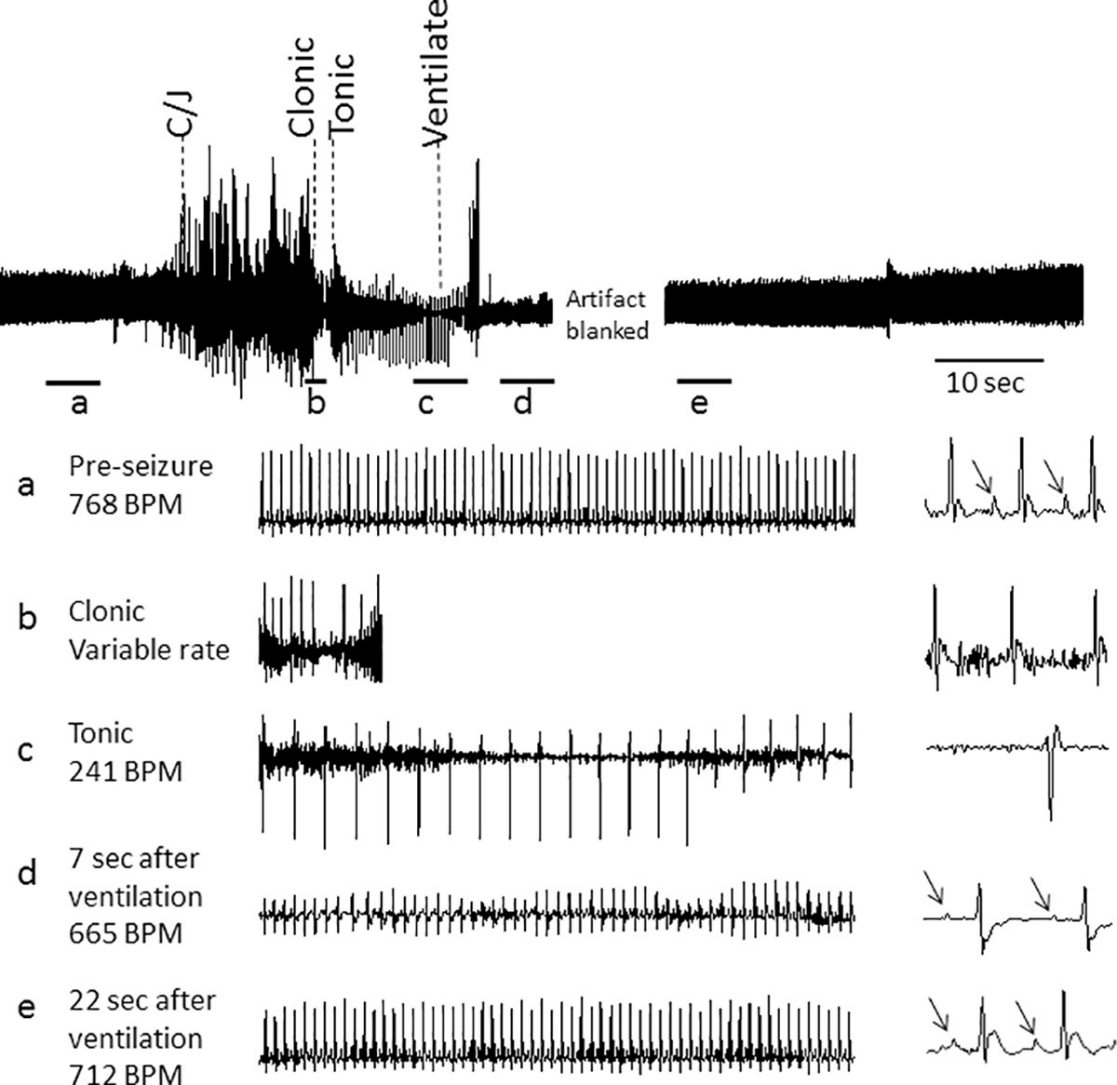
ECG reverts to normal sinus rhythm following resuscitation. The figure shows ECG recording in response to the auditory stimulus through the period of resuscitation. Expanded traces below show 5 sec (**a**, **c-e**) or 1 sec (**b**) during pre-seizure, clonic, tonic phases and two periods following resuscitation; measured heart rate is indicated on the left. During the tonic phase cardiac rhythm is no longer a SA nodal rhythm as indicated by the lack of the P-wave and the slow ventricular rate (**c**). Expanded 200 msec traces on the far right show that nodal rhythm returns during resuscitation. Note reappearance of P-wave associated with QRS complex in **d** and **e** and the shortening in the PR-interval between d and e as the RR-interval decreases, characteristic of a return to nodal rhythm; arrows denote P-waves. PR interval was: (a) 27.5±0.8, (d) 32±1.1, (e) 27.8 ± 0.5 msec.

The ECG was recorded simultaneously with respiration in 6 mice during seizure in response to the acoustic stimulus. In all six of these mice, respiration ceased during the clonic phase; for illustrative purposes, Figs 2 and 3 show only ECG recordings. The RR-interval was followed through the sequence of events from the control period prior to the stimulus through the tonic period. While electrical signals related to the muscular activity during wild running, and to a lesser extent during the C/T phase of the seizure, interfered with the ECG recording, it was possible to follow changes in the RR-interval during most of the recording period. An example of raw data from a mouse that was not resuscitated is provided in Fig 2A. Expanded regions (1 sec; a, b, c) are provided below. As expected, pre-seizure data shows a P-wave linked to the QRS complex. In this example, the RR-interval decreased from 80 ms (Fig 2A, a) to 75 ms prior to the clonic phase. Although the clonic period was short, 2–3 sec, in all mice it was possible to confidently detect the QRS complex at the beginning of the clonic period but, because of the baseline noise, not the P-wave (Fig 2A, b). The RR-interval during the early clonic period (first 1 sec) increased to 87 ms as compared to the pre-seizure level of 80 ms. However by the late tonic phase, when baseline noise was sufficiently reduced, it was clear that the atrial P-wave was absent. The QRS wave, which had the appearance of a shifting ventricular rhythm, increased to an RR-interval of 250 ms (Fig 2A, c). A record of the RR-interval for successive intervals from the pre-stimulus period with an RR interval of 80 ms to a post-ictal interval >600 ms is shown in Fig 2B. In this mouse, which was not resuscitated, the RR interval continued to decay exhibiting an irregular and weak electrical signal that reached an RR interval >1000 ms after approximately 3 more minutes. This was typical of the three mice that were not resuscitated.

To quantify seizure-induced changes in RR-interval, we averaged the RR-intervals (1 sec periods) for pre-seizure, early clonic, early tonic and late tonic intervals and converted the values from RR-interval to heart rate for all 6 mice in this group (Fig 2C). Average heart rate decreased precipitously as the seizure progressed through the clonic and tonic phases from a pre-seizure value of 771±11 to 252±16 bpm near the end of the tonic phase. Thus, the changes in the ECG occur at the same time as the terminal apnea, ie, during the clonic phase.

Three of the six mice were subjected to resuscitation. Fig 3 provides an example of the response in the ECG to the stimulus in one mouse and its recovery with resuscitation. Selected regions of the ECG were expanded in a-e to show the increase in RR-interval in clonic and tonic phases of the seizure. The lack of P-wave driving the QRS is evident during the tonic phase (Fig 3C), and continued during the early resuscitation period, but abruptly returned 7 sec after initiation of ventilation (Fig 3D). The average time for return of the P wave after initiation of ventilation was 8.7 ± 1.3 sec (n = 3).

**Fig 4:**
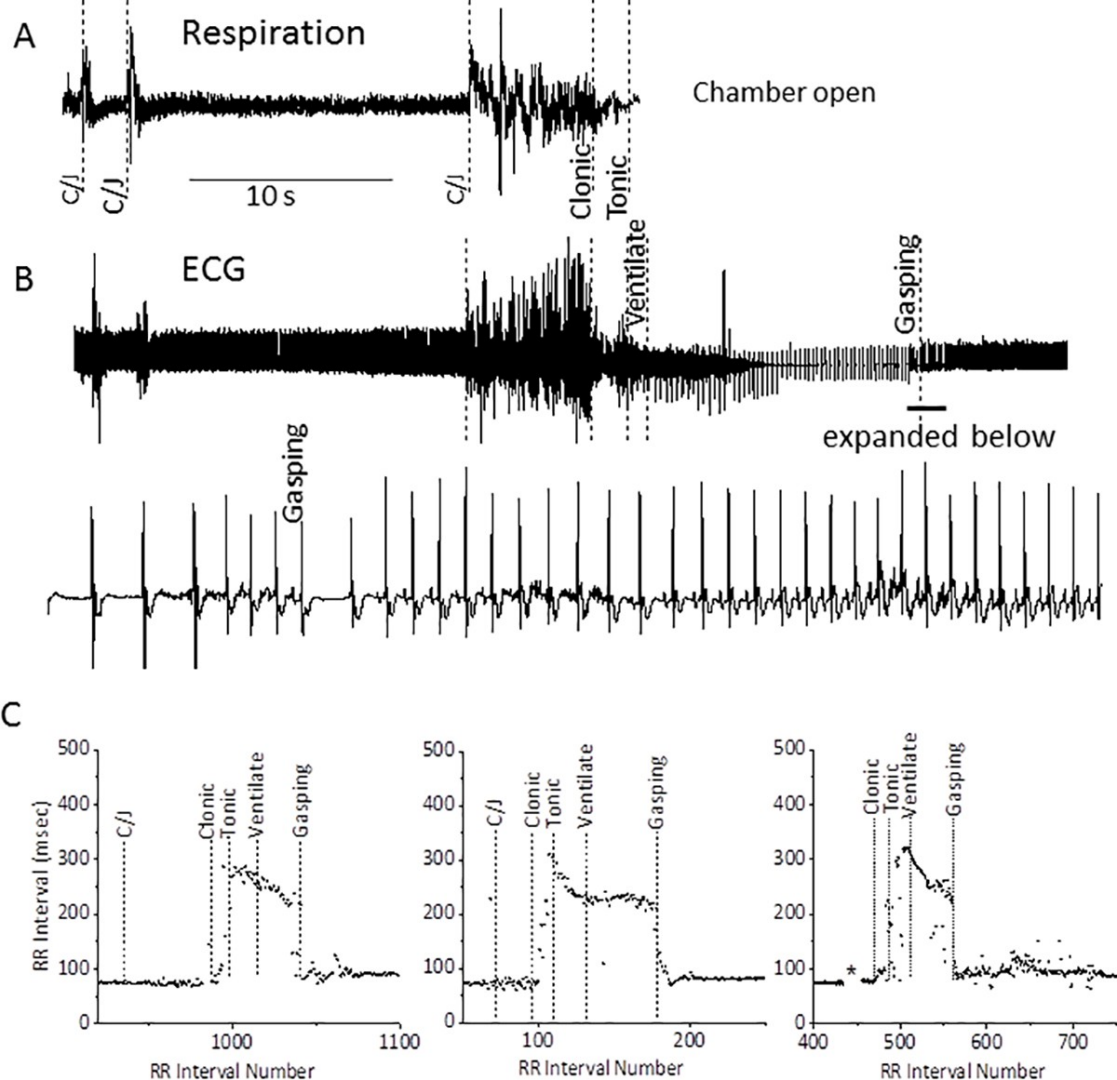
Return of normal cardiac rhythm with mechanical ventilation is immediate and coincides with gasping as captured in video recording. **A-B:** The figure shows the respiratory response to seizure (**A**) with simultaneous ECG recording (**B**). Normal cardiac rhythm is lost rapidly during the clonic phase when respiration ceases, and returns rapidly upon recovery of independent breathing in response to mechanical ventilation as shown in the expanded trace; the point where gasping begins, as seen in the video recording, is indicated. **C**: Tachograms of three different mice capturing the RR-intervals from pre-seizure to recovery. Note the rapid increase in RR-interval occurring with the onset of the clonic phase of the seizure, and the abrupt return to an SA nodal rhythm with ventilation. The asterisk in **c** (far right panel) indicates periods of circling and jumping which interfered with quantification of the RR-interval during these periods.

During the experimental procedure and in subsequent analysis of video recordings, we noted that the mice showed exaggerated gasping as they progressed from complete apnea to unassisted respiration. The relationship between gasping and the reappearance of the P-wave and sinus rhythm is shown in Fig 4. As seen in Fig 4, panel A, respiration again terminated during the clonic phase. During the tonic phase the chamber was opened and artificial ventilation initiated. Within 12 sec, the mouse responded to ventilation with gasping and an abrupt return to a nodal driven cardiac rhythm as shown by the P-waves associated with the QRS complex (Fig 4B). The time courses of the change in the RR-interval obtained from the 3 mice rescued by mechanical ventilation are individually shown in tachograms (Fig 4C). The abrupt change from normal sinus rhythm to pronounced bradycardia between the clonic and tonic phases and the equally abrupt return to normal sinus rhythm during resuscitation is clearly evident in these three mice.

**Fig 5:**
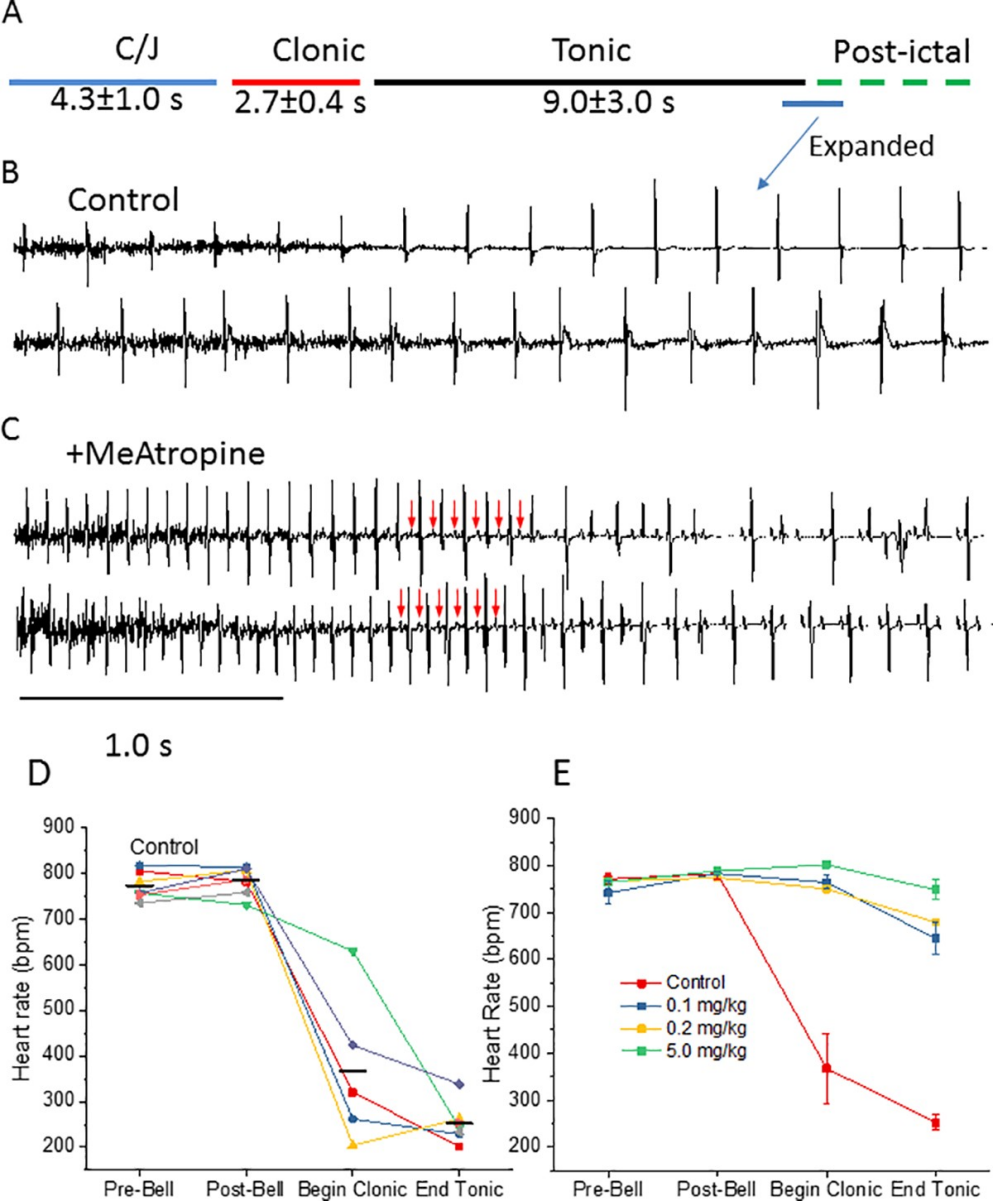
Parasympathetic blockade by methylatropine prevents bradycardia during seizure and in the immediate post-ictal period. **A:** The figure shows the timeline for the various phases of the auditory seizure for 7 mice. **B:** Representative ECG records from 2 mice encompassing the end of the tonic and early post-ictal period. Note the slowed ventricular rate and the absence of P-waves. **C:** Representative ECG records from 2 mice treated with 5mg/kg methylatropine prior to initiation of seizure. The traces show the presence of atrial P-waves in the tonic/early post-ictal period. **D-E:** RR intervals converted to heart rate are shown immediately before the auditory stimulus (*Pre-Bell*), immediately following the stimulus (*Post-Bell*), during the clonic phase (*Begin Clonic*), at the end of the tonic phase (*End Tonic*). **D:** The change in heart rate for individual mice in the absence of methylatropine is shown. Note the rapid decrease in heart rate in the clonic period with a further decrease by the end of the tonic period. Mean values are provided by the black horizontal bars (n = 7, but heart rate at the beginning of the clonic period was obscured by muscle activity for 2 of the 7 control mice). **E:** Heart rate is shown for mice control (red), and mice pre-treated with 0.1 mg/kg (blue, n = 3), 0.2 mg/kg (yellow, n = 2), or 5.0 mg/kg (green, n = 6) methylatropine. For further analysis, the data of the two low concentrations of methylatropine were combined for comparison with control and with the 5mg/kg methylatropine-treated groups using repeated measure ANOVA followed by the Bonferroni post hoc test. There was no significant difference among the three groups prior to the stimulus, whereas both methylatropine groups were significantly different from control, but not from each other, in the clonic and tonic phases (p<0.05).

To investigate the role of the cardiac vagal innervation on cardiac rhythm, in particular on the SA nodal rhythm during the clonic/tonic seizure, 6 mice were given 5 mg/kg ip of the peripheral muscarinic receptor blocker, methylatropine, 20 minutes prior to seizure initiation. Note that the seizure phenotype was identical between controls and methylatropine-treated mice; i.e, seizure induction by the auditory stimulus, 1–2 cycles of circling and jumping followed by loss of posture, limb extension, respiratory arrest and death. The effects of muscarinic blockade by methylatropine on the ECG is shown in Fig 5. The baseline heart rate did not differ significantly between control mice and those that received 5 mg/kg methylatropine (771 ± 11 vs 765 ± 8 bpm, ANOVA, see legend to Fig 5), consistent with the mouse heart rate driven primarily by the sympathetic nervous system [10]. As discussed above, the electrical noise in the baseline ECG produced by muscle contraction obscures the P-wave in the clonic phase and early tonic phase. However, in the presence of methylatropine, as the noise subsides at the end of the tonic phase, a P-wave is clearly present driving a QRS complex (Fig 5C, *red arrows*). This contrasts with the results in controls (Fig 5B) in the absence of muscarinic blockade, where P-waves are absent at the end of the tonic phase (the lack of P-wave in control is also evident in Fig 2A c, and Fig 3C). In 4 of 6 methylatropine-treated mice, resuscitation was not employed. These mice died, and as in control, the ECG deteriorated as the post-ictal period progressed. Heart rates obtained from the RR intervals during pre-ictal and clonic/tonic phases from control mice and methylatropine-treated mice are compared in Fig 5D and 5E. In the absence of the muscarinic receptor blocker, the heart rate falls sharply in the clonic phase and declines to a value of 252 ± 16 bpm at the end of the tonic phase (p<0.05 compared to the baseline value, ANOVA). In mice pre-treated with 5 mg/kg methylatropine however, the rate increased slightly during the clonic phase, but by the end of the tonic phase is only reduced from a baseline value of 765 ± 8 to 748 ± 21 bpm (not significant, p>0.05, ANOVA). Similar results were obtained on mice pre-treated with 0.1 (n = 3) and 0.2 mg/kg (n = 2) methylatropine (Fig 5E). It is important to note that in the presence of methylatropine, a P-wave was present and continued to be seen throughout the immediate post-ictal period, but was often not associated with a QRS-complex as seen near the end of the traces shown in Fig 5C. The dissociation of the P-wave from the QRS complex is indicative of AV block, and was evident throughout the post-ictal period in all methylatropine-treated mice that were allowed to die. In the two mice that were resuscitated, an atrial driven QRS continued to recovery. Thus, the primary effect of methylatropine was the preservation of the P-wave. Muscarinic blockade did not prevent the subsequent seizure-induced AV block, bradycardia, or the establishment of a ventricular escape rhythm. Furthermore, methylatropine did not prevent respiratory arrest during the clonic phase, did not prevent death, and did not limit our ability to resuscitate the mice following seizure.

## Discussion

Several explanations for the rapid death that follows a seizure in SUDEP have been proposed. The majority of clinical observations of SUDEP patients support a central inhibition of respiration during seizure that leads to reduced pO_2_ with subsequent inhibition of cardiac rhythm [2,3]. However, it has also been proposed that the seizure itself initiates a strong cardiac vagal driving force inhibiting cardiac function [9–11]. A combination of the two would indicate a more general central disruption of autonomic function. A possible cause for this autonomic disruption may involve central spreading depression reported in another SUDEP model [15].

In this study we were primarily interested in understanding the changes in cardiac rhythm, and its relationship to alterations in respiratory function, during and immediately after the C/T seizure, and during the resuscitation period. In the experiments where the mice were not resuscitated, we were less interested in asystole *per se* (defined as complete loss of electrical activity) which can be preceded for several minutes by very weak ECG signal and a variable rate of <60 bpm. Asystole undoubtedly occurs long after the mouse has ceased to have a functional cardiac output.

The major finding of the present study is the simultaneous rapid suppression in both respiration and normal cardiac rhythm during the clonic phase of the seizure. Both respiration and heart rate are suppressed in the clonic phase, and by the tonic phase respiration has ceased and the SA node no longer drives cardiac contraction. We reasoned that the rapidity of this response, which is complete within 2–4 sec, suggests both are mediated by central mechanisms. Under normal conditions respiratory rhythm is centrally controlled with modification from a variety of inputs based on metabolic demands. During the C/T seizure respiration abruptly ceases presumably through seizure-activated pathways that block the generation of the central respiratory rhythm. To address origin of the bradycardia and accompanying loss of P-wave driven cardiac rhythm that accompanies the respiratory inhibition, we administered the peripheral muscarinic receptor blocker methylatropine prior to initiating seizure. In the presence of muscarinic blockade, heart rate remained elevated during the tonic phase and during the early postictal periods, and atrial P-waves were present driving the QRS complexes, demonstrating that parasympathetic outflow to the heart during seizure was responsible for the loss of SA nodal rhythm. Indeed, in a previous study, we found that high frequency bilateral stimulation of the vagus nerves in anesthetized mice, produced profound bradycardia with intermittent SA and/or AV nodal block and a ventricular rhythm [10], essentially identical to the results observed in the present study during seizure. However, it is also possible that hypoxemia and/or hypercapnia may contribute to cardiac rhythm disturbances during the C/T seizure. Although both the loss of sinus rhythm during the clonic phase and the abrupt return of sinus rhythm during mechanical resuscitation closely coincide with respiratory changes, the pO2 and pCO2 are undoubtedly very different at these two points in time. Furthermore, several studies in mice have demonstrated that the initial response to hypoxia, ≤12% O_2_, is a reflex increase in heart rate followed by a decrease with no loss of P-wave over several minutes [16–19]. Hypercapnia (5%-6% CO_2_) decreases heart rate, but does not eliminate SA nodal activity [19,20]. Thus, it seems unlikely that alterations in blood gases *per se* contribute to the rapid changes in heart rhythm during C/T seizure.

It is important to note that although P-waves are preserved in DBA/1 mice pretreated with methylatropine, the apnea continues and leads to death if resuscitation is not rapidly provided. A recent study using a mouse model of Dravet syndrome showed that C/T seizures are followed by a sequence of cardiorespiratory changes essentially identical to those observed in the DBA/1 mouse [21]. However, in Dravet mice, seizure-induced apnea, bradycardia, and death can be prevented by high doses of muscarinic receptor antagonists (including methylatropine), a result that appears to be related to the ability of high concentrations of these drugs to cross the blood brain barrier in sufficient quantity to block central muscarinic receptors. Thus, it seems possible that the central mechanisms of seizure-induced respiratory dysfunction are different in Dravet versus DBA/1 mice or that the permeability characteristics of the blood brain barrier are different in these two mouse models.

As noted in *Results*, methylatropine did not prevent seizure-induced AV block and the associated bradycardia. This result suggests that, at least in the mouse, parasympathetic outflow primarily controls SA nodal pacemaker cells, but may have little or no effect on seizure-induced depression of atrioventricular conduction. In this regard, it is possible that AV conduction is influenced by some other mechanism that is unaffected by muscarinic receptor blockade. Recent studies have shown that stimulation of the type 3 sphingosine 1-phosphate receptor (S1P3) by sphingosine 1-phosphate or receptor agonists causes complete AV block that is unaffected by atropine, but completely reversed by a S1P3 antagonist or prevented by knockout of the S1P3 receptor [22]. Sphingosine 1-phosphate is thought to play a cardio-protective role in ischemia/reperfusion injury in the heart [23]. The role of sphingosine 1-phosphate signaling in seizure-induced bradyarrhythmias remains to be determined.

One of the major observations from the MORTEMUS study [3] was the finding that SUDEP in humans was associated with cardiorespiratory dysfunction during the post-ictal period, and that the terminal apnea always preceded the terminal asystole. This is also true in the DBA/1 mice. However, as discussed above, terminal asystole is not a reasonable endpoint for evaluating cardiac function in these mice as cardiac function has been compromised much earlier as indicated by the weak infrequent ECG signal before actual asystole occurs. In the DBA/1 mouse studied here, seizure-associated cardiac and respiratory dysfunction in the C/T phase always occurred simultaneously and recovery of both respiration and heart rate during resuscitation were likewise, closely matched in time. As discussed above, gasping behavior during resuscitation and the near simultaneous reappearance of the P-wave in the ECG recordings suggest a common central mechanism for both autonomic dysfunction and recovery. In this regard, understanding how mechanical ventilation and the neural pathways leading to immediate recovery could provide important clues for the development of neuro-therapeutics for SUDEP.

## Supporting information

S1 Data Set This file contains the values behind the reported means and standard error, and for the construction of figures. (XLSX)
